# Polyhydramnios associated with congenital bilateral vocal cord paralysis: A case report

**DOI:** 10.1097/MD.0000000000031630

**Published:** 2023-02-03

**Authors:** Myeong Gyun Choi, Yoon Ha Kim, Jong Woon Kim, Tae Young Kim, Seo Yeong Park, Hee Young Bang

**Affiliations:** a Department of Obstetrics and Gynecology, Chonnam National University Medical School, Gwangju, Korea.

**Keywords:** bilateral, congenital, polyhydramnios, vocal cord paralysis

## Abstract

Polyhydramnios may develop when the fetus cannot swallow amniotic fluid or the amount of fetal urine increases. Occasionally, unpredictable fetal abnormalities can be diagnosed postnatally. Bilateral vocal cord paralysis in the fetus may cause polyhydramnios, which could be related to impaired prenatal swallowing.

**Patient concern:**

A 36-year-old multipara underwent an emergent cesarean section because of polyhydramnios and active labor at 35 + 5 weeks of gestation and gave birth to a girl.

**Diagnosis:**

The neonate cried feebly and exhibited cyanosis as well as very weak response to stimuli. Chest retraction and stridor were observed. Laryngoscopic examination revealed no movement in both the vocal cords, and bilateral vocal cord paralysis was diagnosed.

**Interventions:**

When the baby was 40 days old, she underwent tracheostomy to alleviate the persistent stridor and oral feeding difficulties.

**Outcomes:**

She was discharged at the age of 60 days while in the tracheostomy state.

**Lessons:**

Securing the airway of neonates with bilateral vocal cord paralysis, tracheoesophageal fistula, or muscular dystrophy, which can be detected after delivery in pregnant women with idiopathic polyhydramnios, is important. Therefore, pregnant women with idiopathic polyhydramnios must be attended to by experts, such as neonatologists, anesthesiologists, or otolaryngologists, who can secure the airway.

## 1. Introduction

Polyhydramnios could be caused when the fetus cannot swallow amniotic fluid or the amount of fetal urine increases.^[1–3]^ It may be idiopathic or associated with a variety of fetal diseases, such as structural abnormalities, genetic disorders, fetal anemia, twin-to-twin transfusion syndrome, neuromuscular disorder, and infection. Maternal diabetes may also cause polyhydramnios.^[4]^

Idiopathic polyhydramnios is associated with no findings other than a prenatal rise in amniotic fluid. While the etiology of polyhydramnios may not be understood at birth, unpredictable fetal abnormalities, such as muscular dystrophy, can occasionally be diagnosed.^[5,6]^

Vocal cord paralysis refers to immobility of the vocal cord. While unilateral vocal cord paralysis is common, bilateral vocal cord paralysis (BVCP) is rare. The causes of BVCP include scarring, iatrogenisis, malignancy, central nervous pathology, systemic disease, and birth trauma; it may also be idiopathic.^[7,8]^ Vocal cord paralysis is an important cause of respiratory compromise in neonates.^[9]^ BVCP especially, often presents with severe airway obstruction leading to stridor or respiratory distress, and up to 73% patients require tracheostomy.^[10]^ It can also cause feeding difficulties in neonates.^[11]^ Therefore, being prepared for treatment of the newborn immediately after delivery is necessary.

We report the case of a neonate with congenital BVCP whose mother had polyhydramnios, and no fetal anatomical deformities were detected prenatally.

## 2. Case report

A 36-year-old multipara visited the hospital at 34 + 2 weeks of gestation with increased amniotic fluid index. The ultrasonographic examination revealed no abnormal fetal findings. The amniotic fluid index was 29, suggesting polyhydramnios. Laboratory tests, including viral study, showed no specific findings. The local hospital’s routine prenatal checkups, including aneuploidy screening test, ultrasonography, and screening for gestational diabetes mellitus, also revealed no specific findings.

Subsequently, the patient presented at the hospital with regular uterine contractions at 35 + 5 weeks of gestation. She underwent an emergent cesarean section and gave birth to a girl. The neonatal birth weight was 2200 g, and the Apgar scores at 1, and 5 minutes were 5 and 8, respectively. The blood acid-base status was 6.989.

The neonate cried feebly and exhibited cyanosis and very weak response to stimuli. Chest retraction and stridor were also observed. Hence, nasal continuous positive airway pressure was applied immediately. However, the saturation pulse oxygen level decreased to 75% and the chest retraction worsened. Therefore, endotracheal intubation was performed, and a surfactant was administered.

**Figure 1.**
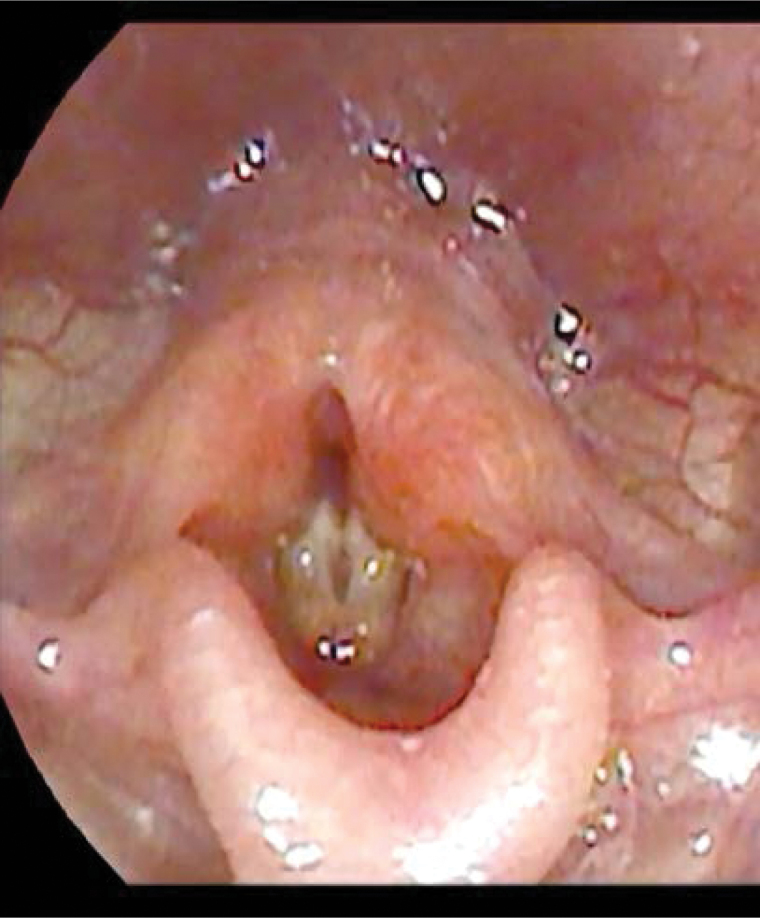
Laryngoscopic examination. It shows no movement in both vocal cords.

Chest radiography revealed decreased diaphragm motion; therefore, ultrasonographic and fluoroscopic examinations were conducted. Nevertheless, no specific findings were noted. The stridor persisted; hence laryngoscopic examination was performed; it revealed no movement in both the vocal cords, and BVCP was diagnosed (Fig. 1).

Owing to the persistent stridor and oral feeding difficulties, the baby underwent a tracheostomy at the age of 40 days and was discharged at the age of 60 days while in the state of tracheostomy. The chromosomal examination, including microarray analysis, did not show any abnormality.

## 3. Discussion

The incidence of polyhydramnios is generally 1% to 2% and it may be diagnosed by checking the amniotic fluid volume using ultrasonography.^[12–15]^ The single, deepest pocket larger than 8 cm or amniotic fluid index greater than 24 cm indicates polyhydramnios.^[16,17]^ Depending on the amount of amniotic fluid, it can be classified into mild, moderate, and severe types.

After diagnosis, thorough sonographic evaluation is required to detect fetal congenital abnormalities. The probability of fetal malformation detection increases with rise in the amniotic fluid volume.^[12,13]^ Furthermore, fetal genetic studies should be considered if congenital abnormalities are detected or severe polyhydramnios is diagnosed.^[18]^ Despite conducting these examinations, 20% of anomalies in neonates are detected postnatally. Sometimes, even conditions including cleft palate, cardiac septal defect, and tracheoesophageal fistula are not diagnosed prenatally.^[19]^

If the unidentified fetal abnormalities are critical enough to require treatment immediately after birth, thorough preparation is required, and BVCP may be one such condition. If a fetus has BVCP, polyhydramnios that occurs probably in relation with impaired prenatal swallowing can also be detected.^[20]^

Vocal cord paralysis is a common cause of neonatal stridor.^[21]^ However, the incidence of BVCP is rare (0.75 cases/million births annually).^[22]^ Clinical presentation is variable, ranging from mild inspiratory stridor to catastrophic airway compromise.^[23]^

BVCP often presents with stridor, feeding difficulties, and other severe symptoms, such as cyanosis and apnea. Fiberoptic laryngoscopy, which can be performed even in very small children, can aid in the diagnosis. The natural history of BVCP depends on its etiology, including trauma, neurologic disorders (e.g., Arnold–Chiari malformation, hydrocephalus, and cerebral palsy), hypoxia, as well as iatrogenic (e.g., related to intubation or surgery) and idiopathic causes.^[24]^

A retrospective review of idiopathic BVCP cases showed that of the 26 neonates, 19 (73%) underwent endotracheal intubation immediately after birth. Among them, 14 (74%) could not be extubated; therefore, they underwent tracheostomy at a median age of 62 days. Spontaneous recovery occurred in 17 patients (65%) of who 9 had undergone tracheostomy, at a mean age of 25 months.^[25]^ In our case, the baby did not recover spontaneously and is currently in the tracheostomy state (age, 4.5 years).

Considering the frequency of endotracheal intubation is high immediately after birth in such cases, being prepared for it is necessary at the time of delivery. Furthermore, securing the airway in neonates with tracheoesophageal fistula or muscular dystrophy, which can be detected after delivery in pregnant women with idiopathic polyhydramnios, is important. In neonates with severe respiratory distress, failure to perform endotracheal intubation at the appropriate time can lead to irreversible brain damage due to hypoxia. Therefore, for pregnant women with idiopathic polyhydramnios, experts, such as neonatologists, anesthesiologists, or otolaryngologists, who can secure the airway must be available.

## 4. Conclusion

Clinicians should note that undetected fetal problems may be encountered in pregnant women with idiopathic polyhydramnios postnatally. The participation of specialists who can secure the airway and ensure proper treatment of the neonates with severe respiratory problems is necessary.

## Author contributions

MGC, JWK, YHK, TYK, SYP, and HYB organized the order of the visits of this patient and interpreted the results. All authors approved the final manuscript as submitted and agree to be accountable for all aspects of the work.

**Conceptualization:** Jong Woon Kim, Yoon Ha Kim.

**Data curation:** Tae Young Kim, Hee Young Bang.

**Investigation:** Seo Yeong Park.

**Project administration:** Myeong Gyun Choi.

**Supervision:** Jong Woon Kim.

**Writing – original draft:** Myeong Gyun Choi.

**Writing – review & editing:** Myeong Gyun Choi, Jong Woon Kim, Yoon Ha Kim.

## References

[1] Harding R Bocking AD Sigger JN. Composition and volume of fluid swallowed by fetal sheep. Q J Exp Physiol. 1984;69:487–95. 6473692 10.1113/expphysiol.1984.sp002835

[2] Pritchard JA. Deglutition by normal and anencephalic fetuses. Obstet Gynecol. 1965;25:289–97. 14268007

[3] Pritchard JA. Fetal swallowing and amniotic fluid volume. Obstet Gynecol. 1966;28:606–10. 5332288

[4] Abele H Starz S Hoopmann M. Idiopathic polyhydramnios and postnatal abnormalities. Fetal Diagn Ther. 2012;32:251–5. 22760013 10.1159/000338659

[5] Dorleijn DM Cohen-Overbeek TE Groenendaal F. Idiopathic polyhydramnios and postnatal findings. J Matern Fetal Neonatal Med. 2009;22:315–20. 19085623 10.1080/14767050802531870

[6] Touboul C Boileau P Picone O. Outcome of children born out of pregnancies complicated by unexplained polyhydramnios. BJOG. 2007;114:489–92. 17309542 10.1111/j.1471-0528.2006.01258.x

[7] Benninger MS Gillen JB Altman JS. Changing etiology of vocal fold immobility. Laryngoscope. 1998;108:1346–50. 9738754 10.1097/00005537-199809000-00016

[8] Benjamin JR Goldberg RN Malcolm WF. Neonatal vocal cord paralysis. NeoReviews 2009;10:e494–501.

[9] Ryan MA Upchurch PA Senekki-Florent P. Neonatal vocal fold paralysis. NeoReviews. 2020;21:e308–22. 32358144 10.1542/neo.21-5-e308

[10] Bower CM Choi SS Cotton RT. Arytenoidectomy in children. Ann Otol Rhinol Laryngol. 1994;103:271–8. 8154768 10.1177/000348949410300403

[11] Miyamoto RC Parikh SR Gellad W. Bilateral congenital vocal cord paralysis: a 16-year institutional review. Otolaryngol Head Neck Surg. 2005;133:241–5. 16087022 10.1016/j.otohns.2005.02.019

[12] Hill LM Breckle R Thomas ML. Polyhydramnios: ultrasonically detected prevalence and neonatal outcome. Obstet Gynecol. 1987;69:21–5. 3540761

[13] Dashe JS McIntire DD Ramus RM. Hydramnios: anomaly prevalence and sonographic detection. Obstet Gynecol. 2002;100:134–9. 12100815 10.1016/s0029-7844(02)02013-6

[14] Biggio JR Jr Wenstrom KD Dubard MB. Hydramnios prediction of adverse perinatal outcome. Obstet Gynecol. 1999;94:773–7. 10546727 10.1016/s0029-7844(99)00370-1

[15] Pri-Paz S Khalek N Fuchs KM. Maximal amniotic fluid index as a prognostic factor in pregnancies complicated by polyhydramnios. Ultrasound Obstet Gynecol. 2012;39:648–53. 21898637 10.1002/uog.10093

[16] Reddy UM Abuhamad AZ Levine D: Fetal Imaging Workshop Invited Participants*Fetal Imaging Workshop Invited Participants*. Fetal imaging: executive summary of a joint Eunice Kennedy Shriver National Institute of Child Health and Human Development, Society for Maternal-Fetal Medicine, American Institute of Ultrasound in Medicine, American College of Obstetricians and Gynecologists, American College of Radiology, Society for Pediatric Radiology, and Society of Radiologists in Ultrasound Fetal Imaging workshop. Obstet Gynecol. 2014;123:1070–82. 24785860 10.1097/AOG.0000000000000245

[17] Dashe JS Pressman EK Hibbard JU; Society for Maternal-Fetal Medicine (SMFM). Electronic address: pubs@smfm.org. SMFM Consult Series #46: evaluation and management of polyhydramnios. Am J Obstet Gynecol. 2018;219:B2–8. 10.1016/j.ajog.2018.07.016 30048635

[18] Boito S Crovetto F Ischia B. Prenatal ultrasound factors and genetic disorders in pregnancies complicated by polyhydramnios. Prenat Diagn. 2016;36:726–30. 27247190 10.1002/pd.4851

[19] Salik I Winters R. Bilateral vocal cord paralysis. In: StatPearls [Internet]. Treasure Island, FL: StatPearls Publishing 2022. 32809687

[20] Brotelande C Leboucq N Akkari M. Isolated neonatal bilateral vocal cord paralysis revealing a unilateral medullary defect: a case report. BMC Pediatr. 2018;18:351. 30413155 10.1186/s12887-018-1329-y PMC6234783

[21] de Jong AL Kuppersmith RB Sulek M. Vocal cord paralysis in infants and children. Otolaryngol Clin North Am. 2000;33:131–49. 10637348 10.1016/s0030-6665(05)70211-5

[22] Murty GE Shinkwin C Gibbin KP. Bilateral vocal fold paralysis in infants: tracheostomy or not? J Laryngol Otol. 1994;108:329–31. 8182321 10.1017/s0022215100126672

[23] Daya H Hosni A Bejar-Solar I. Pediatric vocal fold paralysis. Arch Otolaryngol Neck Surg. 2000;126:21–5. 10.1001/archotol.126.1.21 10628706

[24] Thorpe RK Kanotra SP. Surgical management of bilateral vocal fold paralysis in children: a systematic review and meta-analysis. Otolaryngol Head Neck Surg. 2021;164:255–63. 32689890 10.1177/0194599820944892 PMC10042623

[25] Lesnik M Thierry B Blanchard M. Idiopathic bilateral vocal cord paralysis in infants: case series and literature review. Laryngoscope. 2015;125:1724–8. 25448341 10.1002/lary.25076

